# Fostering Effective Early Learning (FEEL) through a professional development programme for early childhood educators to improve professional practice and child outcomes in the year before formal schooling: study protocol for a cluster randomised controlled trial

**DOI:** 10.1186/s13063-016-1742-1

**Published:** 2016-12-19

**Authors:** Edward Melhuish, Steven J. Howard, Iram Siraj, Cathrine Neilsen-Hewett, Denise Kingston, Marc de Rosnay, Elisabeth Duursma, Betty Luu

**Affiliations:** 1 Early Start and School of Education, University of Wollongong, Wollongong, NSW Australia; 2 Department of Education, Oxford University, Oxford, UK; 3 Department of Psychological Sciences, Birkbeck, University of London, London, UK; 4 Institute of Education, University College London, London, UK; 5 Department of Education, University of Sussex, Brighton, UK

**Keywords:** Early childhood education and care, Professional development, In-service training, Relational and intentional pedagogy, Child development, Preschool education, Intervention, Language development, Numeracy, Self-regulation

## Abstract

**Background:**

A substantial research base documents the benefits of attendance at high-quality early childhood education and care (ECEC) for positive behavioural and learning outcomes. Research has also found that the quality of many young children’s experiences and opportunities in ECEC depends on the skills, dispositions and understandings of the early childhood adult educators. Increasingly, research has shown that the quality of children’s interactions with educators and their peers, more than any other programme feature, influence what children learn and how they feel about learning. Hence, we sought to investigate the extent to which evidence-based professional development (PD) – focussed on promoting *sustained shared thinking* through quality interactions – could improve the quality of ECEC and, as a consequence, child outcomes.

**Methods/design:**

The Fostering Effective Early Learning (FEEL) study is a cluster randomised controlled trial for evaluating the benefits of a professional development (PD) programme for early childhood educators, compared with no extra PD. Ninety long-day care and preschool centres in New South Wales, Australia, will be selected to ensure representation across National Quality Standards (NQS) ratings, location, centre type and socioeconomic areas. Participating centres will be randomly allocated to one of two groups, stratified by centre type and NQS rating: (1) an *intervention* group (45 centres) receiving a PD intervention or (2) a *control* group (45 centres) that continues engaging in typical classroom practice. Randomisation to these groups will occur after the collection of baseline environmental quality ratings. Primary outcomes, at the child level, will be two measures of language development: verbal comprehension and expressive vocabulary. Secondary outcomes at the child level will be measures of early numeracy, social development and self-regulation. Secondary outcomes at the ECEC room level will be measures of environmental quality derived from full-day observations. In all cases, data collectors will be blinded to group allocation.

**Discussion:**

This is the first randomised controlled trial of a new approach to PD, which is focussed on activities previously found to be influential in children’s early language, numeracy, social and self-regulatory development. Results should inform practitioners, policy-makers and families of the value of specific professional development for early childhood educators.

**Trial registration:**

Australian New Zealand Clinical Trials Registry (ACTRN) identifier ACTRN12616000536460. Registered on 27 April 2016. This trial was retrospectively registered, given the first participant (centre) had been enrolled at the time of registration.

**Electronic supplementary material:**

The online version of this article (doi:10.1186/s13063-016-1742-1) contains supplementary material, which is available to authorized users.

## Background

There is widespread consensus that attendance in early childhood education and care (ECEC) is likely to confer a benefit on children [1]. Nonparticipation in ECEC programmes places children at a developmental disadvantage both academically and socially [2, 3]. Furthermore, national surveys [4] and longitudinal research [5] show the enduring benefits that *high-quality* ECEC provides into adolescence and beyond [1]. Indeed, there is strong evidence from independent sources that the benefits of ECEC are moderated by the quality of provision [1].

Recent initiatives in Australia have begun to recognise the short- and long-term benefits of investing in ECEC. The National Quality Framework (NQF) and Early Years Learning Framework (EYLF) [6, 7], for instance, have identified structural and process quality indicators that are important for enhancing child outcomes. These state and national initiatives include measures to improve adult-to-child ratios, introduction of minimum qualifications, regulation based on nationally recognised standards of practice and improvements in curriculum and reporting requirements. This implementation of nationally consistent quality standards is a significant transformation, and a move toward ensuring high-quality educational experiences for children and workforce professionalisation.

In recognition of the important role that ECEC plays in the lives of young children, the New South Wales (NSW) Government has introduced initiatives to ensure that all children have universal access to early education in the year before formal schooling, which starts at 5 years of age. Over recent years, the number of children participating in ECEC services across Australia has increased by 39.5% from 2004 to 2012 [8, 9].

While ECEC attendance in NSW is moving toward a universal provision, there remain questions about the prevalence of high-quality ECEC. Recently, positive long-term associations were documented between ECEC attendance and a range of learning outcomes in national assessments at 8 years of age (i.e. numeracy, reading and spelling) [10]. However, closer inspection of these results showed that the greatest benefits were for children whose preschool teacher held a degree or diploma qualification [10]. Consistent with such findings, the Early Years Workforce Strategy 2012–2016 recognises the importance of a skilled workforce in ensuring ‘the delivery of high-quality ECEC services’ [11]. Nevertheless, the Australian ECEC context continues to be plagued by significant variations in educators’ qualifications. As of January 2014, only ECEC centres with more than 25 places are required to have a full-time, degree-qualified educator. A further 50% of staff needs to be working toward a diploma-level qualification, with the remainder only holding basic certification.

Given this variability in ECEC educators’ training, alternative models, such as ongoing professional development (PD), need to be examined for increasing pedagogical knowledge and improving quality in early childhood educational practice. Increasing child participation in ECEC means that the quality of many young children’s experiences and opportunities depends on the skills, dispositions and understandings of the adult ECEC workforce [12–15].

### The quality of ECEC, child outcomes and professional development

The quality of ECEC is multidimensional, encompassing the physical ECEC environment, the educational curriculum, staff training and qualifications, child-staff ratios, group sizes, staff turnover and interpersonal relationships. Yet, many studies have been discrepant in their conceptualisation of quality and its impact on children’s outcomes. Recent large-scale literature reviews by Melhuish et al. [3] and Siraj and Kingston [16] have concluded, based on international evidence, that the following characteristics of ECEC quality are particularly important for enhancing children’s development:

1. Adult-child interaction that is responsive, affectionate and readily available
2. Well-trained staff who are committed to their work with children
3. A developmentally appropriate curriculum with educational content
4. Ratios and group sizes that allow staff to interact appropriately with children
5. Supervision that maintains consistency in the quality of care
6. Staff development that ensures continuity, stability and improving quality
7. Facilities that are safe, sanitary and accessible to parents
8. Working with families, sharing educational goals and supporting early home learning environments

Many studies have recognised that while physical resources are necessary for quality ECEC, the most important ingredient for quality provision is the quality of the staff who work with the children and families [17]. Cooke and Lawson [18] reported that improving the quality of ECEC and learning outcomes for children required a highly skilled workforce. Increasingly, research has shown that some of the strongest predictors of child outcomes pertained to the quality of adult-child and child-child interactions. Children’s interactions with educators and their peers, more than any other programme feature, are seen as determining what the children learn and how they feel about learning [13, 19–21].

A review and meta-analysis [22] concludes that there is ample evidence that providing sector-specific qualifications and PD for educators improves children’s learning and wellbeing. Evidence has also accrued on the particular value of interactions supporting *sustained shared thinking* (SST). The term sustained shared thinking was originally coined from research considering components of excellent practice in the Effective Provision of Preschool Education (EPPE) study in England [23]. Since this pioneering study, SST has been widely used in many Early Years Frameworks across the world. The EPPE project’s findings, including SST, influenced development of the Australian EYLF [24], as well as England’s Early Years Foundation Stage [25]. However, the practices associated with SST have been found lacking in many ECEC settings [13, 20, 23, 26]. Hence, there is a need to develop staff capacity for fostering interactions that contain SST, as well as other types of interaction that similarly foster language development, critical thinking, self-regulation and social development.

In line with this thinking, a PD programme has been developed that specifically addresses, among other things, the enhancement of staff interactions with children in ways that the available evidence suggests should foster children’s development, especially in the areas of language development, self-regulation, early numeracy and social development. These child outcomes are particularly important because they have been consistently linked to children’s longer-term development in terms of educational achievement and social adjustment in Australia [27, 28], the UK [29], USA [30] and China [31].

### Aims of the study

The main objective of this study is to evaluate whether a PD programme seeking to enhance the quality of ECEC interactions, compared to routine practice, can enhance ECEC quality and child outcomes. The primary outcomes at the child level will be two measures of language development – verbal comprehension and naming vocabulary – as these may be particularly sensitive to environmental changes that may occur from the PD programme [1]. Secondary outcomes at the child level will include other central aspects of child development, notably early numeracy, social development and self-regulation. Secondary outcomes at the centre level will be changes in two environmental rating scales focussing on: (1) curriculum content, concept development and pedagogy and (2) interactional quality through relational and intentional pedagogy. It is hypothesised that the PD intervention will have a positive effect on the identified child outcomes and ECEC room-level environmental quality ratings. If supported, this would provide evidence that improvements in professional practice are mediating child outcomes.

## Methods/design

### Study design

**Fig. 1:**
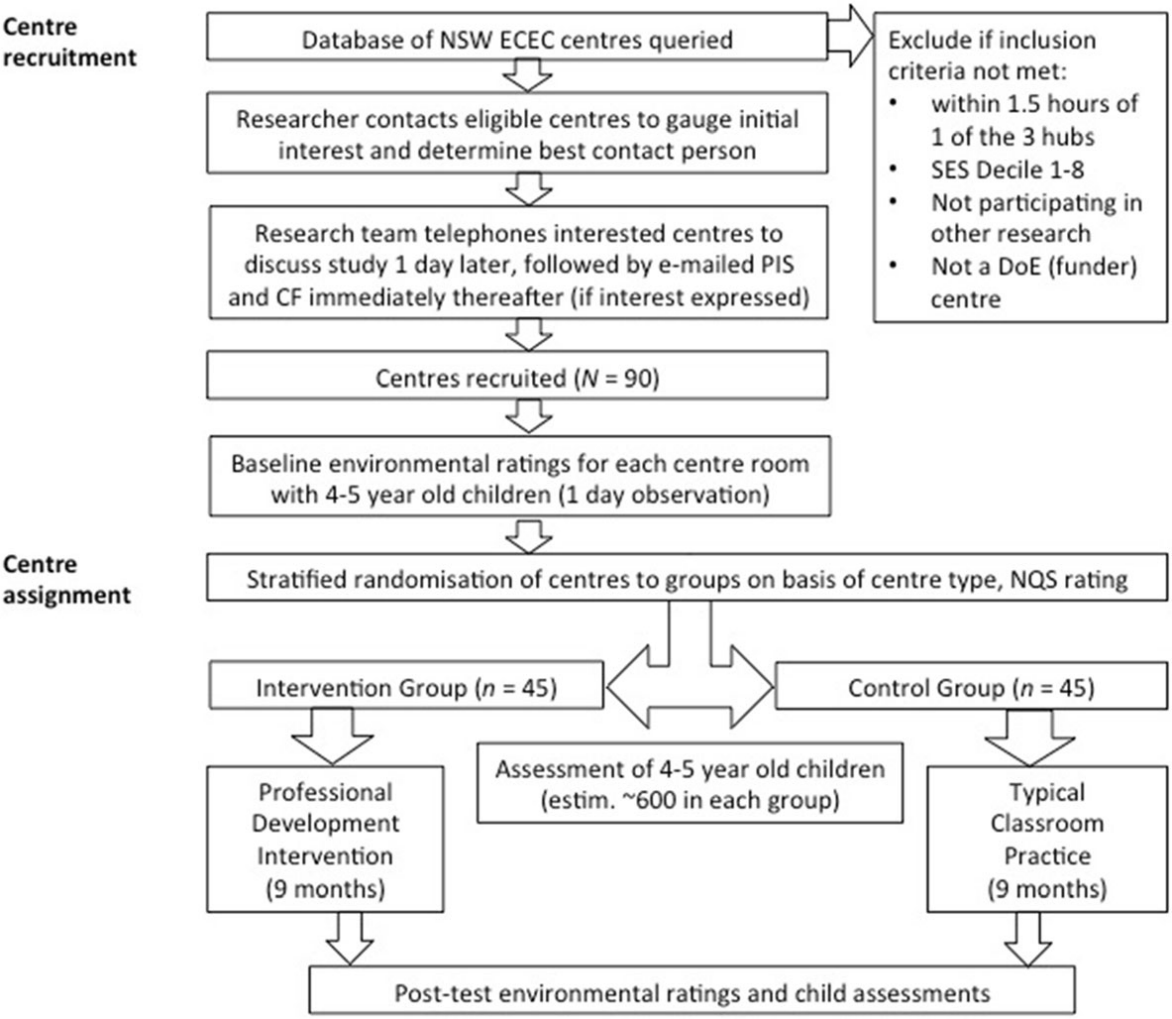
Flow diagram of the stages of the FEEL study

The study employs a clustered randomised controlled trial design. Ninety ECEC centres in NSW, Australia, will be recruited to ensure representation across National Quality Standards (NQS) ratings (Working Towards, Meeting, Exceeding), location (metro, regional), centre type (long-day care and preschool) and socioeconomic areas (as based on the Socio-Economic Indexes for Australia; SEIFA). The sample will ensure representation across these variables, but is not intended to be fully representative of the population (see ‘Centre characteristics and recruitment’ section below). Stratified random assignment of centres to control and intervention groups will occur after the collection of baseline environmental ratings. Once collected, participating centres will then be stratified by centre type and NQS rating and randomly allocated to one of two groups: (1) the *intervention* group (*n* = 45 centres) receiving the PD intervention or (2) the *control* group (*n* = 45 centres) that will continue engaging in typical classroom practice. Fieldworkers, blinded to group allocation, will then conduct baseline child assessments early in the following year. The 9-month PD intervention will occur throughout much of 2016. Post-intervention child assessments and environmental quality ratings will occur again in late-2016 to evaluate any changes as a result of the intervention, relative to control. A flowchart depicting the sequence of recruitment, intervention and assessment for FEEL is shown in Fig. 1. An outline of Standard Protocol Items: Recommendations for Interventional Trials (SPIRIT) time points and actions for the FEEL study is shown in Table 1 (see Additional file 1 for the trial’s SPIRIT Checklist).

**Table 1:** Standard Protocol Items: Recommendations for Intervention Trials (SPIRIT) table

	Study period				
	Enrolment	Allocation	Post allocation		
Time point	Pre intervention	Time (*t*_0_)	Centre baseline (*t*_1a_)	Child baseline (*t*_1b_)	Post intervention (*t*_2_)
Enrolment					
Eligibility screen	X				
Initial centre contact	X				
Centre informed consent	X				
Allocation		X			
Recruitment of children and parent informed consent		X			
Intervention					
PD intervention					X
Regular practice (control)					X
Assessments					
Child assessments					
DAS Verbal Comprehension				X	X
EYT Expressive Vocabulary				X	X
DAS Early Number Concepts				X	X
Early Numeracy Scales				X	X
Strengths & Difficulties (SDQ)				X	X
Child Self-Regulation (CSBQ)				X	X
Centre-level assessments					
ECERS-E Environmental Rating			X		X
SSTEW Environmental Rating			X		X

*PD* professional development, *DAS* Differential Ability Scales, *EYT* Early Years Toolbox, *SDQ* Strengths & Difficulties Questionnaire, *CSBQ* Child Self-Regulation and Behaviour Questionnaire, *ECERS-E* Early Childhood Environment Rating Scale – Extended, *SSTEW* Sustained Shared Thinking and Emotional Wellbeing scale

### Centre characteristics and recruitment

An initial list of ECEC centres in NSW, Australia (*N* = 348) will be examined for potential inclusion. Criteria for the potential inclusion of centres will be: (1) being within 1.5 h of one of the three study hubs, (2) being within socioeconomic (SEIFA) deciles 1–8 (thereby excluding very privileged areas), (3) not participating in other research and (4) not being a Department of Education (study funder) centre. A selection of 90 centres for initial approach will be made on the basis of representation across NQS ratings (i.e. approximately equal numbers of Working Towards, Meeting, Exceeding), service type (i.e. two thirds long-day care, one third preschool), location (i.e. approximately equal numbers of metro and regional centres) and socioeconomic areas (i.e. deciles 1–8, according to SEIFA Advantage and Disadvantage indices, with at least one third of the sample derived from areas of known deprivation). A backup list of centres will be created to supplement recruitment if initial approach is unsuccessful.

Centres will be invited to participate in the study first by an initial call to gauge interest and identify the best contact for follow-up. A member of the research team will then make a follow-up call to interested centres to more fully explain the study and, if appropriate, obtain a contact email address to send Participant Information Sheets and Consent Forms for review. Participating centres will be those that meet the inclusion criteria and return a signed Director Consent Form to participate in the study. The first 90 ECEC centres that respond positively to the invitation and give written consent will take part in the study.

### Randomised allocation of centres

The study will adopt a cluster randomised controlled trial design; participants (i.e. centres and children) will be assigned to the control or intervention groups randomly by cluster (centre). Randomisation will not occur until after: (1) recruitment of centres is complete and (2) initial baseline environment ratings are complete. As such, those involved in recruitment of centres will be unaware, at time of recruitment, to which group centres will be allocated. Stratified randomisation of centres (cluster) will be conducted using centre type and NQS rating as core stratification variables. This randomisation will also be evaluated to ensure comparable socioeconomic status (SES), environmental rating and location profiles between the two groups, which subsequent analyses will confirm.

### Child characteristics and recruitment

Early in the year following centre recruitment, and preceding the intervention, children in the year before formal schooling (4–5 years of age) will be recruited from participating centres. This involves disseminating Participant Information Sheets and Consent Forms, via the centre, to parents or legal guardians of suitably aged children. Participating children will be those who meet the age-inclusion criteria, returned a signed Parental Consent Form to participate in the study and themselves provide verbal assent to participate. Ninety centres are expected to yield a sample of approximately 1200 4–5-year-old children on whom child assessments will be conducted. There are no further exclusion criteria for child participation.

### Outcome measures and procedures

All measures will be administered at baseline and again after the 9-month intervention period (post-test). The battery of child measures was selected to include outcomes that are important for school readiness (i.e. literacy, numeracy, self-regulation, social development). Such outcomes have also been established by previous research as having a foundational role in child development. In total, the child outcome measurements involve 40–50 min of direct assessment per child (split into two sessions) and 10 min of educator time per child (i.e. approximately 3.3 h of educator time per centre) at each data collection time point. In all cases, child assessments will be carried out by a trained fieldworker in a quiet area of the child’s ECEC centre. All environmental quality ratings will also be conducted by highly trained observers through a 1-day observation of each preschool room in participating centres. All observers will need to achieve a rigorous standard of inter-rater reliability with a highly experienced trainer/observer, as indexed by: an intra-class correlation in ratings > .70; a mean difference in ratings < .75; and at least 80% of item ratings within 1 point. In all cases, the researchers involved in collecting baseline and outcome data will be blinded to each centre’s group allocation.

Primary child-level outcomes consist of measures of children’s language development: verbal comprehension and expressive vocabulary. Specifically, the Verbal Comprehension subtest of the Differential Ability Scales (retest reliability 0.82; concurrent validity 0.70) [32] is a measure of the receptive language (comprehension) ability of the child, and takes 10–15 min to administer. The Expressive Vocabulary test from the Early Years Toolbox (internal consistency 0.92; concurrent validity 0.60) [33] is an assessment of expressive vocabulary to complement the receptive language measure, and takes 5 min to administer. Secondary outcomes at the child level involve measures of early numeracy, social development and self-regulation. The Early Number Concepts subscale of the Differential Ability Scales (internal consistency 0.89; concurrent validity 0.71) [32] and the Early Numeracy Assessment [34] include numeric concepts of counting, cardinality, number comparison and number combinations (internal consistency 0.74 to 0.79; concurrent validity 0.35 to 0.66). These two assessments each take 10–15 min to complete. The educator-report Child Self-regulation and Behaviour Questionnaire (CSBQ) [33] and Strengths & Difficulties Questionnaire (SDQ) [35] yield subscales of cognitive, emotional and behavioural self-regulation, antisocial and prosocial behaviours, sociability and anxiety/internalising, among others. The CSBQ subscales have internal consistency ranging from 0.74 to 0.89, and concurrent validity in the range of 0.48 to 0.81 [33]. The SDQ subscales have internal consistency in the range of 0.65 to 0.85, and concurrent validity in the range 0.87 to 0.92 [36].

ECEC room-level secondary outcomes involve observational ratings of the quality of provision in centres using the Early Childhood Environment Rating Scale – Extended (ECERS-E) (inter-observer reliability 0.75 to 0.90 for subscales) [37] and the Sustained Shared Thinking and Emotional Wellbeing (SSTEW) scale (inter-observer reliability 0.79 to 0.92 for subscales) [38]. ECERS-E measures the quality of the curricula, environment and pedagogy in language and literacy, maths and number, science and environment, as well as quality related to meeting the needs of diverse students. The SSTEW scale is designed to consider practice that supports children aged 2 to 5 years in developing skills in SST and emotional wellbeing within five subscales: (1) building trust, confidence and independence, (2) social and emotional wellbeing, (3) supporting and extending language and communication, (4) supporting learning and critical thinking and (5) assessing learning and language.

### Professional development intervention

The PD programme is focussed on enhancing the quality of staff interactions and relational and intentional pedagogy with children. The programme, delivered in three distinct phases over 9 months, provides opportunities to observe, discuss, practice and reflect on important attributes of the effective educator’s role, including: engaging in high-quality interactions and SST, developing and extending concepts, and modelling critical and reflective thinking. Links are made to appropriate frameworks including the Australian NQS and the Australian EYLF. Fundamental to each session is evidence-based understandings of how young children learn best. The PD has been designed to support the collective participation of attendees, as well as to promote collaborative working to gain deeper knowledge of leadership, change management, quality improvement and self-assessment. The PD programme covers eight core content areas, delivered across three phases:

1. Research on quality in ECEC and its assessment
2. How high-quality interactions extend children’s development
3. The relevance of self-regulation to children’s educational success
4. The links between early language development and later literacy
5. Mathematical and scientific concept development in the early years
6. Ways to use observation, assessment of practice and planning to improve quality
7. The importance of early home learning and connections across ECEC settings and the home learning environment
8. The relevance of leadership for learning for children’s development and ways to improve it

#### Phase 1: Intensive professional development (week 1 to week 3, delivered at each of three hubs)

A 2-day, intensive, face-to-face training providing: an overview of national and international research; an introduction to relevant environmental quality characteristics; coverage of key concepts and ideas; as well as strategies to foster early language, cognitive, self-regulatory and social development, engagement in high-quality interactions, and work with homes.

#### Phase 2: Follow-up professional development (week 3 to month 3, delivered at each of three hubs)

Five 4-h, half-day, face-to-face sessions, delivered every 2 weeks, beginning 2 weeks after a hub’s completion of phase 1. The sessions include time for reflection, planning and critical analysis, as well as the introduction of knowledge and pedagogical content on areas not covered in phase 1.

#### Phase 3: Model for sustainability (week 3 to month 9)

To promote centre commitment, limit the effects of staff turnover and increase the likelihood of a positive impact, PD support will be provided for the full 9-month intervention through online modules (beginning at the end of phase 1 and continuing for 9 months). Activities and resources, designed to promote staff engagement and establish an online community of educators are contained within modules or *E-books*. Each E-book combines video-streamed content with questions and text, including links to activities and a discussion forum. Staff participation and discussions feed into a learning portfolio, tracking and reflecting how their ideas about pedagogy, children, families and communities have changed. Access to this online environment is provided to all centre staff, not only those attending phases 1 and 2.

#### Session details

All sessions will be delivered at each of three central hubs to ensure that all centres are within 1.5 h of the PD delivery location. Sessions are to be conducted by four of the study’s chief investigators, who are researchers and international experts in early childhood education and care. All sessions will be delivered in a group setting for centres most proximal to that hub.

### Statistical analyses and power

The primary outcomes are changes in child outcomes that will be analysed in multilevel models where a specific intervention-control comparison will be included. Subsequently, we will compare 45 intervention and 45 control centres for environmental quality in order to estimate the effect upon staff behaviour in the centres.

Analyses will be carried out using two different types of dataset:

1. *The intention-to-treat datasets:* data will be analysed based on participants according to the random allocation, irrespective of whether the intervention was or was not entirely or partly taken up
2. *The per-protocol datasets*: because it is possible that participants may not receive the intervention, the intention-to-treat analysis might underestimate the potential efficacy of intervention. A per-protocol analysis will, therefore, be carried out in addition to the intention-to-treat analysis. The per-protocol datasets will include data pertaining to all outcomes, restricted to participants who complied fully or partly with their assigned intervention.

Primary analyses will use the intention-to-treat dataset. Centre and participant characteristics at trial entry will also be tabulated using the intention-to-treat datasets.

The effect of the PD intervention will be considered in two ways. Firstly, we will compare 45 intervention and 45 control centres for environmental quality in order to estimate the effect upon staff behaviour in the centres. ECEC room-level outcomes will be analysed using linear regression models with post-test outcomes as dependent variables and baseline measures as independent variables. Child-level data analyses will control for clustering within centres using multilevel models (hierarchical models or random-effect models), which can account for clustering of repeated assessments within individuals and clustering of individuals within centres. Post-test scores for primary and secondary outcomes will be used as dependent variables, with baselines variables and group membership as independent variables. Multiple imputation methods will be used for missing individual outcome data. If data are normally distributed we will fit linear models. If not, we will categorise outcome data and use longitudinal logistic regression to evaluate the differences between intervention and control groups. Sensitivity analyses will be conducted for all primary and secondary outcomes. Inverse probability weighting will be considered if missing data are more prevalent than expected and/or there is differential attrition between trial arms. Additionally, reasons for the differential attrition will be fully explored. Secondary analyses from explicit hypotheses (e.g. subgroup, including level of deprivation) will be specified in advance in a statistical analysis plan.

In applying power calculations, we took into account that children are clustered in centres.

Conservative power estimates, after adjusting for the clustered (nested) design, indicate that we can detect an effect size as low as 0.17 standard deviation (SD) units with 80% power for all child outcomes. This is based on a conservative estimate of 13 children per centre, yielding an estimated sample size of 1170. For continuous outcomes, these correspond to differences between the means in the treatment and control groups in units of the SD.

Detectable effect sizes depend on:

1. The type I error rate
2. The unit of comparison, i.e. children (*N* = 1170) or centre (*N* = 90)
3. For children, whether measures are independent observations or clustered within centres
4. Where children are assumed to be clustered within centres, how much of the variability is between centres and how much of the variability is between children within centres

For 80% power, this study would be capable of detecting an effect size for the child outcomes of between 0.17 and 0.20 (*p* < .05) or between 0.21 and 0.24 (*p* < .01), depending on the ratio of the SD of children within centres to the SD of centres. This compares with detectable effect sizes of between 0.16 (*p* < .05) and 0.20 (*p* < .01) if the child measures could be assumed to be independent.

Some of the secondary outcomes involve differences between intervention and control groups at the centre level. Contrasting the child-level outcomes, the differences at the ECEC room level would need to be 0.60 SD units for statistical significance. The design is thus less sensitive for detecting room-level differences than for detecting difference at the child level. While sensitivity for detecting room-level differences could be improved by increasing the sample of centres, the cost implications made this impractical.

### Ethical and research governance approval

The study was granted ethical approval by the University of Wollongong Human Research Ethics Committee Social Sciences (HE15/309) on 8 September 2015. Written consent will be obtained from centre directors (for participation and centre observations), educators (who will complete educator-report measures), and children’s parent(s) or legal guardian(s) as a condition for participation. This will include consent for publication of the study results in anonymised aggregate format. As per Consolidated Standards of Reporting Trials (CONSORT) guidelines, the study’s final reporting will follow the CONSORT Statement and its relevant extensions (e.g. cluster trials, nonpharmacological interventions).

### Study timeline

Recruitment of centres commenced in September 2015 and recruitment of children began in February 2016. Figure 1 provides details of the stages of the study. The trial is set to finish in December 2016.

## Discussion

The study is dependent upon the cooperation of the centres recruited to the study, the staff within those centres and the parents of the children in those centres. Hence, substantial efforts have been expended in producing information leaflets for all concerned to explain the study, its aims and the potential benefits of the study for ECEC centres in the future and the children who will use them. A substantial amount of time has been required for meetings and other liaison between the research team and potential centres for recruitment. Thus far, we believe that the necessary groundwork has been made and hope that the study will proceed according to plan. This would mean that the next publication regarding this trial will concern its results, indicating whether there is an impact of the PD programme on child outcomes as a consequence of improved centre staff practice and pedagogy.

While the FEEL study was designed to yield the strongest possible evidence of intervention efficacy, the study is limited in its focus on intervention evaluation and does not, at present, include further qualitative evaluation (e.g. case studies of centres with the greatest and least change, process evaluation, analysis of PD participants’ feedback and comments). This is due to constraints of the available funding, but could be added should funding become available for these important follow-on investigations. Even in lieu of these additions, the results of the FEEL study should inform decision-making about future practice and policy regarding the provision of evidence-based PD to ECEC staff, as well as potentially informing the content of staff training. Such an impact would be of benefit to ECEC centres and their staff, as well as the communities, families and children who utilise these centres.

### Trial status

Recruitment of ECEC centres began in September 2015 and 90 centres have been recruited. Pretest centre environmental ratings have been completed, random assignment to treatment and control groups has been achieved and recruitment of children is ongoing. Pretest child assessments should be complete before the end of May 2016. Professional development for the treatment group has started and will proceed until the end of the year, with post-test child assessments and environmental ratings starting in November 2016.

## Additional file

Additional file 1: SPIRIT Checklist. (DOC 121 kb)

## Abbreviations

ABS: Australian Bureau of Statistics
ACTRN: Australian New Zealand Clinical Trials Registry
COAG: Council of Australian Governments
CSBQ: Child Self-regulation and Behaviour Questionnaire
DEEWR: Department of Education, Employment and Workplace Relations
DfE: Department for Education (UK)
ECEC: Early childhood education and care
ECERS-E: Early Childhood Environment Rating Scale – Extended
EYLF: Early Years Learning Framework
NQF: National Quality Framework
NQS: National Quality Standards
NSW: New South Wales
OECD: Organisation for Economic Cooperation and Development
PD: Professional development
RCT: Randomised controlled trial
SCSEEC: Standing Council on School Education and Early Childhood
SDQ: Strengths and Difficulties Questionnaire
SEIFA: Socio-economic Indices for Areas
SST: Sustained shared thinking
SSTEW: Sustained Shared Thinking and Emotional Wellbeing
